# A stress granule-associated RNA-binding protein FAM120A drives cisplatin resistance in non-small cell lung cancer

**DOI:** 10.1093/jb/mvaf074

**Published:** 2025-12-15

**Authors:** Shunsaku Hayai, Miho M Suzuki, Kenta Iijima, Keiko Shinjo, Yoshiteru Murofushi, Jingqi Xie, Tatsunori Nishimura, Makoto Ishii, Yutaka Kondo

**Affiliations:** Division of Cancer Biology, Nagoya University Graduate School of Medicine, 65 Tsurumai-cho, Showa-ku, Nagoya, Aichi 466-8550, Japan; Department of Respiratory Medicine, Nagoya University Graduate School of Medicine, 65 Tsurumai-cho, Showa-ku, Nagoya, Aichi 466-8550, Japan; Division of Cancer Biology, Nagoya University Graduate School of Medicine, 65 Tsurumai-cho, Showa-ku, Nagoya, Aichi 466-8550, Japan; Laboratory Animal Facilities and Services, Institute of Photonics Medicine, Hamamatsu University School of Medicine, 1-20-1 Handayama, Higashi-ku, Hamamatsu, 431-3192 Shizuoka, Japan; Division of Cancer Biology, Nagoya University Graduate School of Medicine, 65 Tsurumai-cho, Showa-ku, Nagoya, Aichi 466-8550, Japan; Division of Cancer Biology, Nagoya University Graduate School of Medicine, 65 Tsurumai-cho, Showa-ku, Nagoya, Aichi 466-8550, Japan; Division of Cancer Biology, Nagoya University Graduate School of Medicine, 65 Tsurumai-cho, Showa-ku, Nagoya, Aichi 466-8550, Japan; Division of Cancer Biology, Nagoya University Graduate School of Medicine, 65 Tsurumai-cho, Showa-ku, Nagoya, Aichi 466-8550, Japan; Department of Respiratory Medicine, Nagoya University Graduate School of Medicine, 65 Tsurumai-cho, Showa-ku, Nagoya, Aichi 466-8550, Japan; Division of Cancer Biology, Nagoya University Graduate School of Medicine, 65 Tsurumai-cho, Showa-ku, Nagoya, Aichi 466-8550, Japan; Institute for Glyco-core Research (iGCORE), Nagoya University, Furo-cho, Chikusa-ku, Nagoya, Aichi 464-8601, Japan; Center for One Medicine Innovative Translational Research (COMIT), Nagoya University, Furo-cho, Chikusa-ku, Nagoya, Aichi 464-8601, Japan

**Keywords:** cisplatin resistance, FAM120A, MALAT1, non-small cell lung cancer, stress granule

## Abstract

Cisplatin-based chemotherapy is a standard treatment for non-small cell lung cancer (NSCLC), but drug resistance poses a major clinical challenge. Stress-adaptive mechanisms, such as stress granule (SG) formation, are increasingly recognized alternative pathways that facilitate cancer cell survival. Here, we identify the RNA-binding protein, family with sequence similarity 120A (FAM120A), as an SG-associated factor that drives cisplatin resistance in NSCLC. FAM120A expression was markedly elevated in cisplatin-resistant NSCLC cell lines and clinical tumor specimens and was essential for SG formation and cell survival following cisplatin-induced stress. We found that the intrinsically disordered RNA-binding domain of FAM120A is essential for its incorporation into SGs and for its cytoprotective function. Using enhanced cross-linking immunoprecipitation sequencing data and RNA immunoprecipitation-qPCR, we identified the long noncoding RNA, metastasis-associated lung adenocarcinoma transcript 1 as a key FAM120A interacting partner. MALAT1 levels were reduced upon FAM120A depletion, and overexpression of MALAT1 was sufficient to restore cisplatin resistance in these cells. These findings suggest that MALAT1 is an RNA species that is stabilized by FAM120A and involved in the cellular response to chemotherapy. Targeting this regulatory mechanism may offer new therapeutic strategies to overcome cisplatin resistance in NSCLC.

## Graphical Abstract

**Graphical Abstract:**
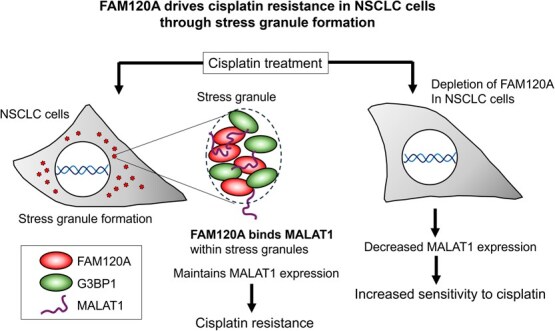

## Abbreviations

ATP7A/B: ATPase copper-transporting alpha and beta
CTR1: Copper transporter 1
eCLIP-seq: enhanced cross-linking immunoprecipitation sequencing
FAM120A: family with sequence similarity 120A
G3BP1: G3BP stress granule assembly factor 1
IDR: intrinsically disordered region
LncRNA: long noncoding RNA; LUAD, lung adenocarcinoma
MALAT1: metastasis-associated lung adenocarcinoma transcript 1
NSCLC: non-small cell lung cancer
RIP: RNA immunoprecipitation
SG: stress granule
TPM: transcripts per million

Lung cancer is the leading cause of cancer-related death worldwide, with non-small cell lung cancer (NSCLC) accounting for approximately 85% of cases *(**1**)*. For decades, cisplatin-based chemotherapy has been a standard treatment for advanced NSCLC. Cisplatin forms DNA crosslinks (primarily intrastrand adducts), thereby disrupting DNA replication and transcription and triggering cell cycle arrest and apoptosis. Despite its efficacy, many patients either fail to respond or develop resistance during treatment, making drug resistance a major obstacle to treatment success *(**2**,*  *3**)*. Cisplatin resistance arises through multiple mechanisms, including reduced drug uptake via transporters, such as copper transporter 1 (CTR1), increased efflux through ATPase copper-transporting alpha and beta (ATP7A/B) or multidrug resistance proteins, enhanced DNA repair activity, evasion of apoptosis and epigenetic reprogramming of stress response pathways *(**3**,*  *4**)*. In addition, recent studies have highlighted the critical role of the tumor microenvironment—including immune cells, stromal components and hypoxia—in shaping the chemotherapeutic response *(**5**)*.

These multifaceted resistance pathways point to the need to explore alternative, stress-adaptive mechanisms beyond the canonical DNA damage response. Among such mechanisms, stress-adaptive cellular structures known as stress granules (SGs) have attracted increasing attention. SGs are membrane-less organelles that arise in the cytoplasm in response to various cellular stressors, including oxidative stress, heat shock and chemotherapeutic agents *(**6**)*. They consist of untranslated mRNAs, RNA-binding proteins, translation initiation factors and small ribosomal subunits *(**7**)*. Among these, G3BP stress granule assembly factor 1 (G3BP1) and G3BP2 are considered key scaffolding components essential for SG assembly *(**8**)*. A hallmark feature of SG-associated RNA binding proteins is the presence of intrinsically disordered regions (IDRs), which facilitate liquid–liquid phase separation and drive dynamic granule formation *(**9**)*. SGs serve as temporary storage sites for untranslated mRNAs and RNA-binding proteins, thereby modulating RNA metabolism *(**8**)*. In cancer, SGs constitute protective hubs that enable tumor cells to survive therapeutic stress. SG formation occurs in many cancer types and has been implicated in resistance to several chemotherapeutic agents, including 5-fluorouracil, sorafenib and bortezomib *(**10**)*. However, the specific molecular players that link SG dynamics to drug resistance remain incompletely understood.

In this study, we focused on an RNA-binding protein, family with sequence similarity 120A (FAM120A), which is involved in the oxidative stress response, microRNA-mediated gene silencing and co-regulation of mTORC1-mediated gene expression *(**11*–*13**)*. Through integrative analysis of cisplatin-resistant NSCLC cell lines and SG-associated proteomic datasets, we identified FAM120A as a SG-associated factor whose expression correlates with cisplatin resistance. We further investigated whether the modulation of SG formation and the maintenance of stress-responsive RNAs by FAM120A explains its ability to promote chemoresistance.

## Materials and Methods

### Cell culture and transfection

A549 cells (RIKEN Cell Bank, Japan) were cultured in DMEM supplemented with 5% fetal bovine serum and 1× Antibiotic-Antimycotic (Gibco, Thermo Fisher Scientific, MA, USA) at 37°C in a humidified incubator with 5% CO₂. A549 cells are a well-established NSCLC model for cisplatin sensitivity *(**14**,*  *15**)* and exhibited reproducible stress granule formation upon cisplatin exposure. Transfections of siRNAs and plasmids were performed using Lipofectamine 3000 (Thermo Fisher Scientific) according to the manufacturer’s instructions. siRNAs and plasmids used in this study are listed in Table S1.

### Western blotting

Cells were lysed in RIPA buffer (50 mM Tris–HCl pH 7.4, 150 mM NaCl, 1% NP-40, 0.1% SDS, 0.5% sodium deoxycholate) supplemented with 1× Complete protease inhibitor cocktail (Roche, Switzerland). Lysates were incubated on ice for 30 min and cleared by centrifugation at 12,000 × *g* for 15 min at 4°C. Protein concentration was determined using a BCA assay (Thermo Fisher Scientific), and equal amounts of protein were separated by SDS-PAGE and transferred onto PVDF membranes (Millipore, Germany). Membranes were blocked with 5% bovine serum albumin in TBS-T (Tris-buffered saline with 0.1% Tween-20) for 1 h and incubated overnight at 4°C with primary antibodies against FAM120A or β-actin (loading control). After washing, membranes were incubated with HRP-conjugated secondary antibodies for 1 h at room temperature. Protein bands were visualized using enhanced chemiluminescence (ECL) reagents (Thermo Fisher Scientific) and imaged with a LAS-4000 system (GE Healthcare, IL, USA). Antibodies used are listed in Table S1.

### SG visualization by immunofluorescence

A549 cells were seeded onto glass coverslips in 24-well plates and transfected as described above. After 24 h, cells were treated with 20 μM cisplatin (cis-diammineplatinum(II) dichloride, Sigma-Aldrich, MO, USA, Cat #P4394) for 24 h to induce SG formation. Following treatment, cells were fixed with 4% paraformaldehyde for 15 min at room temperature and permeabilized with 0.1% Triton X-100 in PBS for 10 min. After blocking with 5% bovine serum albumin in PBS for 1 h, cells were incubated overnight at 4°C with primary antibodies against G3BP1 and FAM120A. After washing, cells were incubated with Alexa Fluor–conjugated secondary antibodies for 1 h at room temperature. Nuclei were counterstained with DAPI. Images were captured with a fluorescent microscope (DMI6000B, Leica, Germany) with 63× objectives. Antibodies used are listed in Table S1.

### Cell viability assay

Cell viability was assessed using Cell Count Reagent SF (Nacalai Tesque, Japan) according to the manufacturer's instructions. A549 cells were seeded in 96-well plates and co-transfected with siRNAs and plasmids as described above. After 24 h, cells were treated with various concentrations of cisplatin (0, 10, or 20 μM) for an additional 48 h. Cell Count Reagent SF was added directly to each well (10 μl per 100 μl medium), and plates were incubated for 1 h at 37°C. Absorbance at 450 nm was measured using a microplate reader (Bio-Rad iMark). Cell viability was calculated relative to untreated control wells and expressed as the mean ± standard deviation (SD) of triplicate measurements.

### Apoptosis assay by flow cytometry

Apoptosis was evaluated using the Annexin V-FITC Apoptosis Detection Kit (Nacalai Tesque, Japan) according to the manufacturer's instructions. A549 cells were seeded in 6-well plates, transfected with siRNAs and treated with cisplatin (20 μM) for 48 h. Cells were harvested, washed twice with cold PBS and resuspended in binding buffer. Cells were then stained with Annexin V-FITC and propidium iodide for 15 min at room temperature in the dark. Stained samples were immediately analysed using a Gallios flow cytometer (Beckman Coulter, CA, USA). The percentage of apoptotic cells (Annexin V-positive) was quantified using FlowJo software (version 10.6.1; BD Biosciences, NJ, USA).

### RNA immunoprecipitation followed by qPCR

RNA immunoprecipitation (RIP) was performed as described previously *(**16**)*. A549 cells were lysed in RIP lysis buffer supplemented with RNase inhibitor (NIPPON GENE, Japan) and protease inhibitor cocktail (Roche, Switzerland). Cell lysates were pre-cleared and incubated with Protein A/G magnetic beads conjugated to either anti-FAM120A antibody or normal rabbit IgG (negative control) at 4°C overnight with gentle rotation. After extensive washing, RNA–protein complexes were eluted and treated with proteinase K, and the RNA was extracted using TRIzol reagent (Thermo Fisher Scientific). Purified RNA was reverse transcribed using PrimeScript RT Master Mix (Takara Bio, Japan), and target RNAs were quantified by qPCR using SYBR Green-based detection. Metastasis-associated lung adenocarcinoma transcript 1 (MALAT1) enrichment was calculated relative to the input. Antibodies and qPCR primers used are listed in Table S1.

### Cancer Cell Line Encyclopedia, The Cancer Genome Atlas and ENCODE dataset analysis

Publicly available datasets were analysed to assess the clinical and functional relevance of FAM120A. Using DepMap (https://depmap.org), protein expression and cisplatin IC_50_ data for NSCLC cell lines were obtained from the Cancer Cell Line Encyclopedia (CCLE), a comprehensive resource that provides genomic, transcriptomic, proteomic and pharmacologic profiles of a large panel of human cancer cell lines *(**17**)*, and differential expression analyses between cisplatin-resistant and -sensitive groups were performed using GraphPad Prism and R. RNA sequencing data for tumor and normal lung tissues, as well as clinical response information, were downloaded from The Cancer Genome Atlas (TCGA) lung adenocarcinoma (LUAD) cohort using the UCSC Xena browser (https://xenabrowser.net).

FAM120A enhanced Cross-Linking Immunoprecipitation sequencing (eCLIP-seq) datasets in K562 cells were obtained from the ENCODE project (https://www.encodeproject.org). Raw FASTQ files were aligned to the human reference genome (hg38), and transcript-level read counts were quantified using Ballgown. Long noncoding RNAs (lncRNAs) were annotated based on GENCODE v39, and read counts were normalized to identify highly enriched FAM120A-bound transcripts. LncRNAs were ranked according to average read abundance across duplicate datasets.

### Statistical analysis

All experiments were performed in at least three biological replicates. Results are shown as mean and the error bars represent SD. Microsoft Excel and GraphPad Prism 9 (GraphPad Software, CA, USA) were used to generate graphs and to perform statistical analysis. *P*-values were calculated using a two-sided Student's *t*-test unless stated otherwise. *P*-values of statistical significance are indicated as ns, *P* > 0.05; ^*^P < 0.05; ^**^P < 0.01; ^***^P < 0.001.

## Results

### FAM120A is upregulated in cisplatin-resistant NSCLC and associates with stress granules

**Fig. 1:**
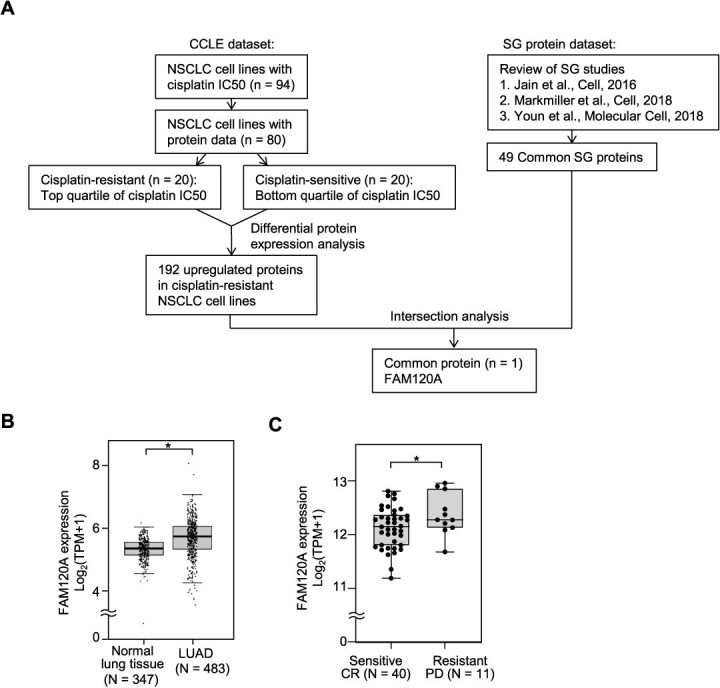
**Identification of FAM120A as a SG protein associated with cisplatin resistance in NSCLC.** (A) Flowchart illustrating the selection of cisplatin-resistant NSCLC cell lines and identification of FAM120A. NSCLC cell lines were classified as cisplatin-resistant or cisplatin-sensitive using CCLE data. Differential expression analysis identified 192 upregulated proteins in resistant cell lines. A review of three studies identified 49 SG proteins. FAM120A was the only protein common to both groups. (B) FAM120A expression levels were compared between LUAD tumor tissues (*n* = 483) and normal lung tissues (*n* = 347) using TCGA data. Expression is shown in transcripts per million (TPM). FAM120A expression was significantly higher in LUAD tumors than in normal lung tissues (*P <* 0.05). (C) FAM120A expression levels were analysed in cisplatin-treated LUAD cases categorized as responders (CR, *n* = 40) or resistant (PD, *n* = 11) based on treatment outcome. Expression is shown in TPM. Each dot represents an individual data point. Whiskers extend to 1.5 × IQR. FAM120A expression was significantly higher in resistant patients compared to responders (*P <* 0.05).

Among 80 NSCLC cell lines with both cisplatin IC_50_ values and protein expression data available in CCLE, we classified the top quartile (*n* = 20) as cisplatin-resistant and the bottom quartile (*n* = 20) as cisplatin-sensitive based on IC_50_ values (Table S2). A differential protein expression analysis was performed to compare these two groups. We identified 192 proteins that were significantly upregulated (*P* < 0.05) in the cisplatin-resistant cell lines compared to the cisplatin-sensitive cell lines (Table S3). To systematically investigate proteins associated with SGs, we conducted a comparative analysis of three studies that focused on SG protein composition *(**18*–*20**)*. Through this analysis, we identified a core set of 49 proteins that was consistently detected across all three datasets (Table S4). Then, we cross-referenced the two lists, identifying proteins that were both upregulated in cisplatin-resistant NSCLC cell lines and known components of SGs. By applying this analytical framework, we prioritized SG-associated proteins with potential involvement in cisplatin resistance and identified FAM120A as the only protein common to both categories (Fig. 1A).

To assess the clinical relevance of FAM120A expression in NSCLC, we analysed RNA sequencing data from TCGA LUAD dataset. FAM120A expression was significantly higher in tumor tissues (*n* = 483) than in normal lung tissues (*n* = 347, *P* < 0.05) (Fig. 1B). We further examined the association between FAM120A expression and cisplatin treatment response in LUAD patients. In cisplatin-treated cases categorized by treatment outcome (*(**21**)*; Table S5), FAM120A expression was significantly higher in resistant patients compared to responders (*P* < 0.05) (Fig. 1C).

### FAM120A is required for stress granule formation and cisplatin resistance

**Fig. 2:**
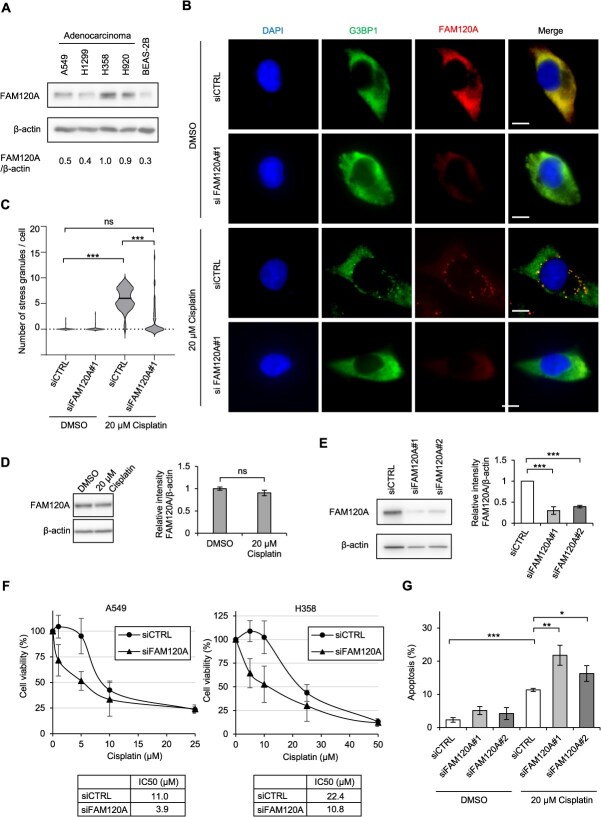
**FAM120A promotes SG formation and cisplatin resistance in NSCLC cells.** (A) Western blot analysis of FAM120A protein levels in NSCLC cell lines (A549, H1299, H358 and H920) and the normal immortalized alveolar epithelial cell line BEAS-2B. β-actin was used as a loading control. (B) Immunofluorescence staining of A549 cells treated with cisplatin (20 μM, 24 h). SGs were visualized using the SG marker G3BP1, and FAM120A was assessed by immunostaining. Co-localization of G3BP1 and FAM120A signals was observed. Nuclei were counterstained with DAPI. Scale bar, 10 μm. (C) Violin plot showing the number of SGs per cell in siCTRL and siFAM120A-treated A549 cells. A total of 100 cells were analysed per condition. Horizontal bars indicate the median. (D) Top: Western blot analysis showing that cisplatin treatment (20 μM, 24 h) did not alter the protein level of FAM120A in A549 cells. β-actin was used as a loading control. Bottom: Quantification of FAM120A protein levels from three independent experiments. Data are presented as mean ± SD. (E) Left: Western blot analysis showing reduced FAM120A protein levels in A549 cells transfected with siRNA targeting FAM120A for 48 h. β-actin was used as a loading control. Right: Quantification of FAM120A protein levels from three independent experiments. Data are presented as mean ± SD. (F) Dose–response viability curves of A549 and H358 cells transfected with siCTRL or siFAM120A and treated with increasing concentrations of cisplatin for 72 h. The table below shows IC_50_ values calculated from the cell viability data. (G) Flow cytometric analysis of apoptosis in A549 cells transfected with siCTRL or siFAM120A for 24 h, followed by treatment with DMSO or cisplatin (20 μM) for an additional 24 h. Apoptosis was assessed by Annexin V staining. Data are presented as mean ± SD. ^*^P < 0.05, ^**^P < 0.01, ^***^P < 0.001 significance determined using Student's *t*-test.

Our analysis revealed that FAM120A protein expression was moderately to markedly elevated in the NSCLC cell lines A549, H1299, H358 and H920, relative to the normal immortalized alveolar epithelial cell line BEAS-2B (Fig. 2A). To determine whether FAM120A is incorporated into cisplatin-induced SGs, we performed fluorescence immunostaining in A549 cells treated with cisplatin (20 μM, 24 h). This treatment induced cytoplasmic SGs, as marked by G3BP1, and FAM120A co-localized with them (Fig. 2B and C). The cisplatin exposure did not affect the overall protein level of FAM120A (Fig. 2D). Depletion of FAM120A with siRNA significantly impaired the formation of cisplatin-induced SGs, as evidenced by a marked reduction in G3BP1-positive cytoplasmic foci (Fig. 2B, C, E), suggesting that FAM120A is required for efficient SG assembly under cisplatin-induced stress conditions. We next examined the sensitivity of A549 cells to cisplatin upon FAM120A depletion. Cell viability assays revealed that the IC_50_ for cisplatin was significantly reduced from 11.0 μM in control siRNA (siCTRL)-treated A549 cells to 3.9 μM in siFAM120A-treated cells (Fig. 2F, left). A similar reduction was observed in H358 cells, where the IC_50_ decreased from 22.4 μM in siCTRL-treated cells to 10.8 μM in siFAM120A-treated cells (Fig. 2F, right). Consistently, flow cytometric analysis of Annexin V-positive cells showed that FAM120A depletion significantly increased cisplatin-induced apoptosis compared to control cells (Fig. 2G).

### The RNA-binding domain of FAM120A is essential for its stress granule localization and cisplatin resistance

**Fig. 3:**
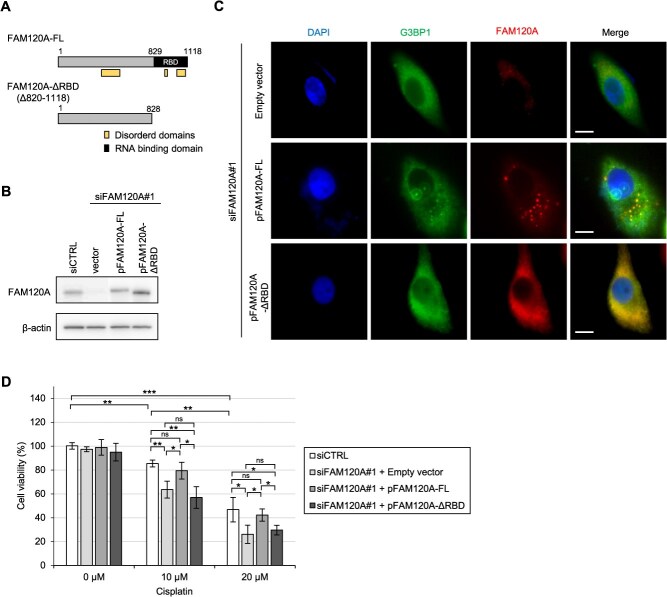
**The RNA-binding domain of FAM120A is required for SG formation and cisplatin resistance.** (A) Schematic representation of full-length FAM120A and the RNA-binding domain (RBD) deletion mutant (ΔRBD). The region labeled “RBD” indicates the RNA-binding domain identified by Tanaka *et al. (**23**)*. The regions annotated as “disordered domains” represent IDRs predicted by MobiDB Lite Consensus Disorder Prediction, retrieved from InterPro. (B) Western blot analysis of A549 cells co-transfected with siFAM120A and either empty vector, FAM120A-FL, or FAM120A-ΔRBD for 48 h. Expression of FAM120A constructs was detected using an anti-FAM120A antibody. β-actin served as a loading control. (C) Immunofluorescence staining of A549 cells co-transfected with siFAM120A#1 and either empty vector, FAM120A-FL, or FAM120A-ΔRBD for 24 h, followed by treatment with 20 μM cisplatin for an additional 24 h. SGs were visualized by G3BP1 immunostaining, and FAM120A localization was assessed in relation to SGs. Nuclei were stained with DAPI. Scale bar, 10 μm. (D) Viability of A549 cells co-transfected with siFAM120A#1 and either empty vector, FAM120A-FL, or FAM120A-ΔRBD for 24 h, followed by treatment with 0, 10, or 20 μM cisplatin for an additional 48 h. Data are presented as mean ± SD, *n* = 3. ^*^P < 0.05; ^**^P < 0.01; ^***^P < 0.001; ns, not significant; two-sided *t*-test.

SGs typically contain IDR-containing RNA-binding proteins that promote phase separation and granule assembly *(**22**)*. Given that FAM120A seems essential for SG formation (Fig. 2B) and that its RNA-binding domain includes IDRs (Fig. 3A), we next investigated whether this domain is required for its incorporation into SGs and its role in promoting cisplatin resistance. To test this, we generated a full-length construct (FAM120A-FL) and a deletion mutant lacking the RNA binding domain (FAM120A-ΔRBD) (Fig. 3A). Western blotting confirmed that both constructs were expressed at comparable levels under conditions of endogenous FAM120A depletion (Fig. 3B). Immunofluorescence analysis of cisplatin-treated A549 cells (20 μM, 24 h) revealed that FAM120A-FL localized to G3BP1-positive SGs, whereas the ΔRBD mutant did not (Fig. 3C). Furthermore, only FAM120A-FL restored cisplatin resistance in FAM120A-depleted cells. This finding indicates that reintroduction of FAM120A confers resistance and that the RNA binding domain is essential for both SG localization and FAM120A-mediated resistance to cisplatin (Fig. 3D).

### MALAT1 binds FAM120A and contributes to cisplatin resistance in a stress-dependent manner

**Fig. 4:**
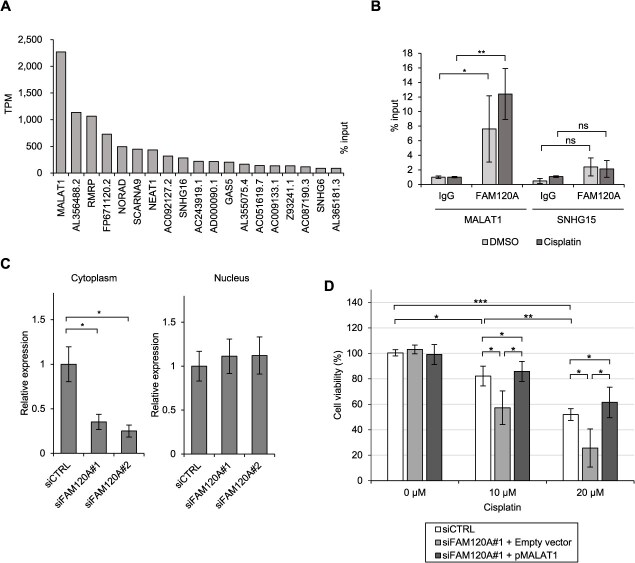
**FAM120A binds to the stress-responsive lncRNA MALAT1 and contributes to cisplatin resistance.** (A) Analysis of ENCODE eCLIP-seq data for FAM120A in K562 cells. lncRNAs bound by FAM120A were ranked by read abundance (transcripts per million, TPM), revealing MALAT1 as the top interactor. (B) Validation of the FAM120A-MALAT1 interaction by RIP-qPCR in A549 cells treated with or without cisplatin (20 μM, 24 h). SNHG15, which showed only minimal binding to FAM120A in ENCODE eCLIP-seq data, was used as a negative control. Relative enrichment of MALAT1 and SNHG15 was quantified by qPCR. Data are shown as mean ± SD, *n* = 3. ^*^P < 0.05; ^**^P < 0.01; two-sided *t*-test. (C) qRT-PCR analysis of MALAT1 expression in cytoplasmic and nuclear fractions of A549 cells transfected with siFAM120A. Data are shown as mean ± SD, *n* = 3. ^**^P < 0.01; two-sided *t*-test. (D) Viability of A549 cells transfected with siFAM120A#1 and either empty vector or MALAT1-expressing plasmid, followed by treatment with 20 μM cisplatin for 48 h. Data are presented as mean ± SD, *n* = 3. ^*^P < 0.05; ns, not significant; two-sided *t*-test.

To identify RNA targets of FAM120A, we analysed publicly available eCLIP-seq datasets from the ENCODE project. Based on the K562 cell dataset, we extracted transcripts bound by FAM120A and focused specifically on lncRNAs, which are known to play context-dependent roles in stress responses and often function within phase-separated compartments *(**24**,*  *25**)*. Among all lncRNAs bound by FAM120A, MALAT1, emerged as the top candidate in terms of read abundance (Fig. 4A). MALAT1 has previously been implicated in lung cancer progression, including invasion and metastasis *(**26**)*. RIP-qPCR analysis in A549 cells validated the interaction between FAM120A and MALAT1, which was further enhanced by cisplatin treatment (20 μM, 24 h) (Fig. 4B). Following FAM120A knockdown, MALAT1 levels were markedly decreased in the cytoplasm, whereas nuclear MALAT1 remained unchanged (Fig. 4C). Furthermore, forced expression of MALAT1 in FAM120A-depleted cells restored cisplatin resistance (Fig. 4D), indicating that MALAT1 mediates FAM120A-dependent resistance to cisplatin-induced cytotoxic stress.

## Discussion

In this study, we identified the RNA-binding protein FAM120A as a critical SG-associated factor that promotes cisplatin resistance in NSCLC. Our data show that FAM120A is upregulated in cisplatin-resistant cells and is essential for SG assembly under cisplatin-induced stress. The RNA binding domain of FAM120A is indispensable for its incorporation into SGs and for conferring resistance to cisplatin-induced apoptosis. Furthermore, we demonstrated a functional interaction between FAM120A and the lncRNA MALAT1, with FAM120A maintaining MALAT1 expression levels. This suggests a novel mechanism whereby SG-associated RNA-binding proteins modulate chemoresistance via RNA regulation.

SGs are increasingly recognized as protective cellular structures that enable cancer cells to survive various stresses, including chemotherapy *(**10**)*. By sequestering pro-apoptotic signaling molecules, SGs transiently inhibit apoptosis and thereby promote cell survival *(**27**,*  *28**)*. Additionally, SGs regulate RNA metabolism by selectively stabilizing transcripts necessary for stress adaptation while suppressing global translation. Key SG components such as G3BP1, TIA-1 and Caprin-1 have been implicated in chemoresistance across multiple cancer types through these selective RNA regulatory functions *(**10**)*.

Our findings extend this model by implicating FAM120A as a pivotal SG component that orchestrates an adaptive response to cisplatin-induced stress. The non-canonical SGs induced by cisplatin, characterized by altered composition and persistence *(**29**)*, may represent specialized structures that sustain chronic stress signaling and therapeutic resistance. The requirement of FAM120A for SG assembly suggests that it plays a role in remodeling SG composition to favor cancer cell survival following chemotherapy.

Although MALAT1 primarily localizes to nuclear speckles under normal conditions, it can redistribute to the cytoplasm under stress, such as hypoxia, inflammation and chemotherapy *(**24**)*. This stress-induced cytoplasmic MALAT1 likely interacts with FAM120A to promote chemoresistance, adding a new dimension to the function of MALAT1 beyond its nuclear roles. Indeed, a recent study demonstrated that cytoplasmic functions of MALAT1 in the brain are both regulated by and involved in modulating synaptic function *(**23**)*.

MALAT1 contributes to cisplatin resistance in NSCLC through diverse mechanisms. As a competing endogenous RNA, it acts as a ‘sponge’ for tumor-suppressive microRNAs such as miR-145 and miR-146a, thereby upregulating targets like KLF4 and BRCA1 that enhance cancer stemness and DNA repair, respectively *(**30**,*  *31**)*. MALAT1 also activates STAT3 signaling, increasing expression of drug efflux transporters MDR1 and MRP1, thereby reducing intracellular cisplatin levels and drug efficacy *(**32**)*. Our study shows that MALAT1 strongly associates with FAM120A and that its stabilization by FAM120A contributes to the survival of cisplatin-treated cells. While we established the critical role of the RNA binding domain of FAM120A in SG localization and chemoresistance, the precise mechanisms by which MALAT1 is stabilized—whether via direct binding or recruitment of protective complexes—remain to be elucidated. Future work should investigate whether other noncoding RNAs are similarly regulated by FAM120A or participate in SG-mediated resistance. Furthermore, validation in additional NSCLC cell lines, particularly FAM120A-high lines such as H358 or H920, would help establish the broader relevance of the proposed mechanism.

Collectively, our results highlight a functional RNA–protein interaction that enhances cell survival, revealing a previously unrecognized layer of SG-mediated chemoresistance in which specific RNA-binding proteins protect noncoding RNAs with stress-modulatory roles. This also suggests that disrupting such RNA-binding proteins-lncRNA interactions could represent a novel therapeutic strategy to sensitize cancer cells to chemotherapy. Given the growing interest in targeting phase-separated compartments therapeutically, FAM120A and its RNA partners offer promising opportunities for intervention.

## Conclusion

In summary, our study identifies the RNA-binding protein FAM120A as a key mediator of cisplatin resistance in NSCLC through its roles in stress granule assembly and stabilization of the lncRNA MALAT1. These findings uncover a previously unrecognized RNA–protein regulatory axis that supports cancer cell survival under chemotherapeutic stress. Targeting FAM120A or its interactions with RNA may provide a novel approach to overcoming drug resistance in lung cancer and other malignancies.

## Supplementary Material

Web_Material_mvaf074
