# Supplementary material for: A stress granule-associated RNA-binding protein FAM120A drives cisplatin resistance in non-small cell lung cancer
Source: J Biochem. 2025 Dec 15;179(2):127–35. doi: 10.1093/jb/mvaf074 (PMC12866663; doi:10.1093/jb/mvaf074)
Supplement: Web_Material_mvaf074 [file web_material_mvaf074.zip › 03_Supplementary Table S1-5newNov.pdf]

Supplementary Table S1. List of reagents used in this study

Plasmids

Name	Description	References / Catalog no.
pFAM120A-FL	This plasmid contains the full-length FAM120A cDNA cloned into the pcDNA backbone.	This study
pFAM120A-ΔRBD	This plasmid is based on pFAM120A-FL and expresses a deletion mutant of FAM120A lacking amino acids 829–1,118.	This study
pMALAT1	Obtained from the RIKEN DNA Bank. It consists of a full-length MALAT1 transcript cloned into the pCMV-SPORT6 backbone.	IRAK134J16 (RIKEN DNA BANK)

siRNAs

Name	Sequence (5'-3') / Catalog no.	Concentration
siCTRL	#4390843 Silencer Select Negative Control #1 siRNA (Thermo Fisher Scientific)	20 nM
siFAM120A#1	UUUCAUAAUCAUAAAAAUCAA	20 nM
siFAM120A#2	CAAACAAAGGCAGAAGGCUCGUCCA	20 nM

qPCR Primers

Name	Sequence (5'-3')	References / Catalog no.
GAPDH-F	ACATCGCTCAGACACCATG	Hs.PT.39a.22214836 (IDT)
GAPDH-R	TGTAGTTGAGGTCAATGAAGGG	
MALAT1-F	GGTGCTACACAGAAGTGGATTC	This study
MALAT1-R	CTCCTCAGTCCTAGCTTCATCA	
SNHG15-F	GTCTTCGGCAGTCTAGTCATC	This study
SNHG15-R	CTCTTCCACTTTGAGACCGTC	

Antibodies

Name	Company	Catalog no.	Dilution	Experiment
Mouse monoclonal anti-FAM120A	abcepta	AW5641	1:2000	WB
Mouse monoclonal anti-β-Actin	Cell signaling Technology	3700	1:1000	WB
Horse anti-mouse IgG, secondary antibody, HRP	Cell signaling Technology	7076	1:2000	WB
Horse anti-rabbit IgG, secondary antibody, HRP	Cell signaling Technology	7074	1:2000	WB
Rabbit monoclonal anti-G3BP	Abcam	ab181150	1:100	IF
Mouse monoclonal anti-FAM120A	abcepta	AW5641	1:100	IF
Goat anti-rabbit IgG, secondary antibody, Alexa Fluor 488	Thermo Fisher Scientific	A11008	1:1200	IF
Goat anti-mouse IgG, secondary antibody, Alexa Fluor 568	Thermo Fisher Scientific	A11004	1:1200	IF
Rabbit polyclonal anti-FAM120A (OSSA)	Bethyl Laboratories	A303-889A	2μg	RIP
Rabbit IgG	MBL	PM035	2μg	RIP

Supplementary Table S2. List of cisplatin-resistant and cisplatin-sensitive NSCLC cell lines selected from the CCLE dataset

Cisplatin-resistant NSCLC cell lines (top quartile of IC50, n = 20)

Study ID	Patient ID	Sample ID	IC50 (μM)
ccle_broad_2019	EKVX_LUNG	EKVX_LUNG	808.5
ccle_broad_2019	NCIH1993_LUNG	NCIH1993_LUNG	314.2
ccle_broad_2019	NCIH2291_LUNG	NCIH2291_LUNG	277.7
ccle_broad_2019	NCIH2444_LUNG	NCIH2444_LUNG	224.9
ccle_broad_2019	NCIH1838_LUNG	NCIH1838_LUNG	212.5
ccle_broad_2019	CALU3_LUNG	CALU3_LUNG	178.0
ccle_broad_2019	NCIH596_LUNG	NCIH596_LUNG	167.0
ccle_broad_2019	LU65_LUNG	LU65_LUNG	158.4
ccle_broad_2019	CAL12T_LUNG	CAL12T_LUNG	148.5
ccle_broad_2019	PC3JPC3_LUNG	PC3JPC3_LUNG	147.5
ccle_broad_2019	NCIH441_LUNG	NCIH441_LUNG	136.8
ccle_broad_2019	NCIH1435_LUNG	NCIH1435_LUNG	124.2
ccle_broad_2019	NCIH2135_LUNG	NCIH2135_LUNG	108.6
ccle_broad_2019	UMC11_LUNG	UMC11_LUNG	103.5
ccle_broad_2019	NCIH292_LUNG	NCIH292_LUNG	94.8
ccle_broad_2019	NCIH727_LUNG	NCIH727_LUNG	93.9
ccle_broad_2019	NCIH1770_LUNG	NCIH1770_LUNG	87.0
ccle_broad_2019	RERFLCSQ1_LUNG	RERFLCSQ1_LUNG	86.9
ccle_broad_2019	NCIH322M_LUNG	NCIH322M_LUNG	85.4
ccle_broad_2019	NCIH2347_LUNG	NCIH2347_LUNG	81.2

Cisplatin-sensitive NSCLC cell lines (bottom quartile of IC50, n = 20)

Study ID	Patient ID	Sample ID	IC50 (μM)
ccle_broad_2019	NCIH661_LUNG	NCIH661_LUNG	9.0
ccle_broad_2019	EMCBAC2_LUNG	EMCBAC2_LUNG	8.8
ccle_broad_2019	HCC827_LUNG	HCC827_LUNG	8.3
ccle_broad_2019	NCIH1651_LUNG	NCIH1651_LUNG	7.9
ccle_broad_2019	NCIH1734_LUNG	NCIH1734_LUNG	7.8
ccle_broad_2019	NCIH1581_LUNG	NCIH1581_LUNG	7.7
ccle_broad_2019	NCIH2023_LUNG	NCIH2023_LUNG	6.9
ccle_broad_2019	NCIH2110_LUNG	NCIH2110_LUNG	6.8

ccle_broad_2019	NCIH1155_LUNG	NCIH1155_LUNG	6.7
ccle_broad_2019	PC14_LUNG	PC14_LUNG	6.0
ccle_broad_2019	201T_LUNG	201T_LUNG	5.9
ccle_broad_2019	JHU028_LUNG	JHU028_LUNG	5.7
ccle_broad_2019	NCIH23_LUNG	NCIH23_LUNG	5.6
ccle_broad_2019	CORL23_LUNG	CORL23_LUNG	5.4
ccle_broad_2019	NCIH460_LUNG	NCIH460_LUNG	5.3
ccle_broad_2019	NCIH1781_LUNG	NCIH1781_LUNG	5.1
ccle_broad_2019	HOP62_LUNG	HOP62_LUNG	4.8
ccle_broad_2019	HOP92_LUNG	HOP92_LUNG	2.8
ccle_broad_2019	ABC1_LUNG	ABC1_LUNG	2.7
ccle_broad_2019	HCC44_LUNG	HCC44_LUNG	2.6

Supplementary Table S3. List of proteins upregulated in cisplatin-resistant non-small cell lung cancer cell lines compared to cisplatin-sensitive cell lines in CCLE dataset

Gene symbol	Mean expression (Sensitive)	Mean expression (Resistant)	Log2 fold change	P-value
MUC20	-2.19	-0.07	2.11	1.10E-04
MARVELD3	-1.37	0.91	2.28	1.16E-03
TMEM143	-0.82	0.25	1.06	1.17E-03
LGR4	-1.18	0.38	1.56	1.22E-03
CRB3	-2.08	0.84	2.93	1.74E-03
GSTT2	-1.28	0.58	1.86	2.38E-03
HK3	-0.46	0.04	0.5	2.52E-03
PON2	0.05	0.86	0.82	2.66E-03
PARVA	-0.45	0.35	0.79	3.17E-03
FMN1	-1.14	0.28	1.41	3.21E-03
PLEKHJ1	-0.47	0.1	0.57	3.54E-03
ARL1	-0.21	0.32	0.53	3.64E-03
ITGA11	-1.01	-0.02	0.98	4.36E-03
PDXK	-0.4	0.42	0.82	4.40E-03
FRY	-0.4	0.15	0.55	4.47E-03
SEC23A	-0.03	0.5	0.53	4.71E-03
DTWD1	-0.64	0.81	1.45	5.23E-03
USO1	-0.28	0.26	0.54	5.48E-03
SLC25A16	0.03	0.61	0.59	5.50E-03
RAB17	-1.05	0.57	1.63	5.53E-03
ABHD17A	-0.1	0.67	0.77	5.81E-03
TBL2	-0.21	0.38	0.59	5.81E-03
GNA12	-0.23	0.2	0.43	5.84E-03
A1CF	-1.55	0.59	2.15	6.26E-03
TMEM120A	-0.35	0.66	1.01	6.37E-03
SLC35A1	-0.21	0.62	0.83	6.48E-03
SLC31A1	-0.31	0.61	0.92	6.88E-03
GNA11	-0.17	0.36	0.53	7.73E-03
IFNGR1	-0.61	0.45	1.06	7.94E-03
ANO1	-1.09	0.21	1.3	8.07E-03
SYPL1	-0.49	0.5	0.99	8.15E-03

TMEM101	-0.18	0.41	0.6	8.21E-03
TMEM106B	-0.05	0.64	0.69	8.43E-03
GASK1B	-1.44	0.85	2.29	8.88E-03
RRAS	-0.2	0.48	0.68	8.92E-03
PON3	-0.16	2.66	2.82	9.73E-03
TJP1	0.26	1.34	1.07	0.0106
IL13RA1	-0.02	0.74	0.76	0.0111
PEX6	-0.23	0.44	0.67	0.0111
ARHGAP18	-0.73	0.26	1	0.0112
PEX1	-0.16	0.48	0.64	0.0113
SLC39A3	-0.67	0.29	0.96	0.0113
CASP10	-1.79	0.63	2.42	0.0118
ZDHHC8	-0.29	0.44	0.73	0.0118
ANXA4	-0.84	-0.09	0.75	0.0121
CAB39	-0.31	0.4	0.71	0.0122
IQGAP1	-0.17	0.51	0.68	0.0123
JAK1	-0.32	0.36	0.68	0.0123
KDELR1	0.03	0.59	0.57	0.0124
CAPN2	-0.37	0.6	0.97	0.0126
UXT	-0.59	0.2	0.79	0.0127
NDFIP1	-0.93	0.5	1.43	0.0132
VAV2	-0.51	0.23	0.74	0.0133
RALB	-0.3	0.3	0.61	0.0135
ERGIC1	-0.4	0.48	0.87	0.0136
MVP	-0.45	0.59	1.04	0.0136
PTTG1IP	-0.26	0.76	1.03	0.014
ACSL5	-0.7	0.5	1.2	0.0145
SEC24A	-0.24	0.4	0.64	0.0145
NIBAN2	-0.55	0.45	1	0.0149
ZDHHC5	-0.37	0.18	0.55	0.015
CCDC71L	-0.24	0.42	0.66	0.0157
CARMIL1	-0.46	0.26	0.72	0.0163
PGM2L1	-0.22	0.72	0.94	0.0164
DHRS7	0.06	0.82	0.76	0.0165
RNF19A	-1.16	-0.17	0.99	0.0166
NMNAT3	-1.82	-0.22	1.6	0.0167

ACAA1	-0.1	0.46	0.56	0.0171
MICAL2	-0.43	0.76	1.19	0.0171
AP3B1	-0.27	0.17	0.44	0.0172
UBR3	-0.16	0.14	0.3	0.0172
ILK	-0.36	0.07	0.43	0.0178
RAC1	-0.24	0.12	0.36	0.0184
SMPDL3A	-0.33	0.56	0.89	0.0187
SLC30A5	-0.13	0.34	0.47	0.0188
CASP8	-0.55	0.22	0.77	0.0189
FAM120A	-0.32	0.21	0.53	0.0191
SLC15A1	-2.05	-0.01	2.04	0.0191
CLK1	-0.39	0.34	0.73	0.0193
PRKAG2	-0.14	0.42	0.56	0.0193
POR	-0.17	0.61	0.77	0.0197
MASP1	-0.96	0.63	1.59	0.0199
PARP4	-0.62	0.29	0.91	0.0201
AKR7A3	-1.37	0.3	1.67	0.0203
DTX2	-0.33	0.57	0.9	0.0216
ANKK1	-0.67	0.2	0.86	0.0221
IL1RAP	-1.13	0	1.13	0.0221
MGAT4A	-0.98	0.75	1.72	0.0221
APPL2	-0.35	0.19	0.54	0.0222
RALA	-0.34	0.22	0.56	0.0224
GRAMD4	-0.45	0.25	0.7	0.0228
ACVR1	-0.57	0.36	0.93	0.023
ACSL1	-0.46	0.34	0.8	0.0231
ABHD17B	-0.23	0.24	0.47	0.0235
SLC35E1	0.04	0.55	0.51	0.0235
GPRIN2	-0.12	0.43	0.55	0.0236
EIF4E2	-0.13	0.13	0.26	0.0241
RHOT1	-0.32	0.15	0.47	0.0249
NDRG1	-0.69	0.32	1.02	0.026
DOP1B	-0.23	0.62	0.84	0.0261
MYO1E	-0.79	0.36	1.14	0.0264
CCM2	-0.36	0.21	0.57	0.0267
MCTP2	-0.53	0.67	1.2	0.0267

CDKN1A	-1.03	0.33	1.36	0.027
CXORF38	-0.45	0.19	0.64	0.0273
C4ORF33	-1.13	0.03	1.16	0.0276
DGCR2	-0.39	0.37	0.76	0.0277
TYK2	-0.44	-0.01	0.43	0.0278
SMURF1	-0.08	0.72	0.79	0.0282
MR1	-0.75	1.32	2.08	0.0283
CRYBG3	-0.37	0.49	0.86	0.0285
LYST	-0.75	-0.02	0.73	0.0286
PCBD2	-0.04	0.48	0.52	0.0287
LGALS3	-0.36	0.94	1.3	0.0288
PHLDA3	-1.16	0.38	1.54	0.0291
PTPRE	-0.47	0.69	1.16	0.0293
CD81	-0.74	0.01	0.75	0.0297
LMBRD1	-0.11	0.57	0.68	0.0298
TRAPPC2	-0.19	0.45	0.64	0.0298
VSTM2L	-0.06	2.05	2.1	0.0305
ME1	-0.47	0.71	1.18	0.0308
MYD88	-0.31	0.58	0.89	0.0309
REEP3	-0.29	0.5	0.78	0.0309
RHOD	-0.7	0.76	1.46	0.0309
TMEM65	-0.32	0.37	0.69	0.0309
REEP5	-0.22	0.38	0.6	0.0312
IGFBP4	-0.16	1.23	1.39	0.0316
YTHDC2	-0.34	0	0.33	0.0321
MGST3	-0.38	0.34	0.72	0.0322
GBP2	-0.86	0.18	1.04	0.0325
AMIGO2	-0.29	0.91	1.21	0.0331
IDH1	-0.15	0.5	0.65	0.0331
NEDD4	0.01	0.51	0.5	0.0334
RHOBTB3	-0.52	0.93	1.45	0.0334
RPS6KC1	-0.34	0.11	0.45	0.0334
TRIM56	0.05	0.39	0.34	0.0336
AGAP1	-0.3	0.17	0.47	0.0348
MTHFS	-0.2	0.35	0.56	0.035
ANKIB1	-0.12	0.21	0.32	0.0357

SURF4	-0.19	0.23	0.42	0.0358
USP54	-0.03	0.48	0.51	0.0358
PAFAH2	0.06	0.69	0.63	0.0359
STS	-0.4	0.69	1.09	0.0361
SCARB2	-0.25	0.45	0.71	0.0364
TRAPPC9	-0.16	0.22	0.39	0.0371
DDB2	-0.42	0.36	0.78	0.0377
TTC39C	-0.56	0.21	0.77	0.0377
CD151	-0.39	0.22	0.61	0.038
TMEM87B	-0.52	0.24	0.76	0.0381
ENDOD1	-0.25	0.37	0.62	0.0387
TGM2	-0.01	1.3	1.31	0.039
WIPI1	-0.66	0.07	0.72	0.0396
WDR11	-0.11	0.16	0.27	0.0398
DENND4C	-0.31	0.03	0.34	0.0401
HMGCR	-0.67	-0.03	0.64	0.0401
TCL1A	-0.98	-0.35	0.64	0.0404
PDXDC1	-0.38	0.29	0.66	0.0407
LONP2	-0.25	0.2	0.45	0.0412
TMEM68	-0.35	0.19	0.54	0.0412
TJP3	-0.2	0.69	0.89	0.0413
ATP11A	0.01	0.6	0.58	0.0418
F2R	-1.53	0.37	1.9	0.0425
NHSL2	-0.77	0.11	0.88	0.0425
SEC24B	-0.18	0.17	0.35	0.0428
SGK3	-0.42	0.74	1.16	0.043
ACOX3	-0.22	0.5	0.72	0.0433
CCSER2	-0.45	0.2	0.65	0.0433
CDIPT	-0.28	0.03	0.32	0.0435
SMPD1	-0.17	0.48	0.65	0.0435
PCYOX1	-0.04	0.46	0.5	0.0437
TXNIP	-0.4	0.62	1.02	0.0444
SCARA3	-1.06	-0.27	0.79	0.0446
SCAMP2	-0.08	0.44	0.52	0.0449
TCF7L2	-0.53	0.04	0.57	0.0451
TXNDC11	-0.14	0.43	0.58	0.0457

SLC2A13	-0.45	0.72	1.17	0.0459
TAOK3	-0.18	0.39	0.57	0.0467
SC5D	-0.37	0.34	0.71	0.0468
TSKU	0.28	0.98	0.7	0.047
PI4KA	-0.33	0.18	0.51	0.0473
VAC14	-0.18	0.33	0.52	0.0474
VKORC1L1	-0.09	0.25	0.34	0.0474
ARHGAP42	-0.36	0.73	1.09	0.0481
CLCN3	-0.43	-0.04	0.39	0.0482
SDC4	-0.49	0.71	1.2	0.0484
DAAM1	-0.44	0.24	0.67	0.0486
LIMS1	-0.4	0.01	0.41	0.0488
LYSMD3	-0.13	0.47	0.59	0.0491
MLANA	-2.09	0.11	2.2	0.0494
ARHGAP35	-0.55	0.07	0.63	0.0496
RAB27A	-0.18	0.67	0.85	0.0497
RHOB	-0.29	0.62	0.91	0.0499

Supplementary Table S4. List of proteins commonly identified in stress granules across three studies

Gene symbol	Protein name
ATXN2	Ataxin 2
ATXN2L	Ataxin 2 Like
CAPRIN1	Cell Cycle Associated Protein 1
CASC3	CASC3 Exon Junction Complex Subunit
CELF1	CUGBP Elav-Like Family Member 1
CSDE1	Cold Shock Domain Containing E1
DAZAP1	DAZ Associated Protein 1
DDX3X	DEAD-Box Helicase 3 X-Linked
DHX36	DEAH-Box Helicase 36
FAM120A	Family With Sequence Similarity 120 Member A
FMR1	Fragile X Messenger Ribonucleoprotein 1
FUBP3	Far Upstream Element Binding Protein 3
FXR1	FMR1 Autosomal Homolog 1
FXR2	FMR1 Autosomal Homolog 2
G3BP1	G3BP Stress Granule Assembly Factor 1
G3BP2	G3BP Stress Granule Assembly Factor 2
HNRNPAB	Heterogeneous Nuclear Ribonucleoprotein A/B
IGF2BP1	Insulin Like Growth Factor 2 MRNA Binding Protein 1
IGF2BP2	Insulin Like Growth Factor 2 MRNA Binding Protein 2
IGF2BP3	Insulin Like Growth Factor 2 MRNA Binding Protein 3
LSM14A	LSM14A MRNA Processing Body Assembly Factor
LSM14B	LSM Family Member 14B
MAGED1	MAGE Family Member D1
NUFIP2	Nuclear FMR1 Interacting Protein 2
PABPC1	Poly(A) Binding Protein Cytoplasmic 1
PABPC4	Poly(A) Binding Protein Cytoplasmic 4
PCBP1	Poly(RC) Binding Protein 1
PRRC2A	Proline Rich Coiled-Coil 2A
PRRC2C	Proline Rich Coiled-Coil 2C
PUM1	Pumilio RNA Binding Family Member 1
PUM2	Pumilio RNA Binding Family Member 2
RBMS1	RNA Binding Motif Single Stranded Interacting Protein 1

RBMS2	RNA Binding Motif Single Stranded Interacting Protein 2
RC3H1	Ring Finger And CCCH-Type Domains 1
STAU2	Staufen Double-Stranded RNA Binding Protein 2
SYNCRIP	Synaptotagmin Binding Cytoplasmic RNA Interacting Protein
TDRD3	Tudor Domain Containing 3
TIAL1	TIA1 Cytotoxic Granule Associated RNA Binding Protein Like 1
TNRC6B	Trinucleotide Repeat Containing Adaptor 6B
UBAP2	Ubiquitin Associated Protein 2
UBAP2L	Ubiquitin Associated Protein 2 Like
UPF1	UPF1 RNA Helicase And ATPase
USP10	Ubiquitin Specific Peptidase 10
YBX3	Y-Box Binding Protein 3
YTHDF1	YTH N6-Methyladenosine RNA Binding Protein F1
YTHDF2	YTH N6-Methyladenosine RNA Binding Protein F2
YTHDF3	YTH N6-Methyladenosine RNA Binding Protein F3
ZC3HAV1	Zinc Finger CCCH-Type Containing, Antiviral 1

The three studies referenced in this table are:

1. Jain et al., 2016, Cell 164, 487-498, DOI: 10.1016/j.cell.2015.12.038;
2. Markmiller et al., 2018, Cell 172, 590-604, DOI: 10.1016/j.cell.2017.12.032;
3. Youn et al., 2018, Molecular Cell 69, 517-532, DOI: 10.1016/j.molcel.2017.12.020.

Supplementary Table S5. Analysis of NSCLC cases from TCGA in this study (Figure 1C)

Sample Id	Group	mRNA Expression of FAM120A*
TCGA-05-4384-01	Resistant	12.274291
TCGA-53-7624-01	Resistant	12.472264
TCGA-53-7626-01	Resistant	12.851771
TCGA-55-6970-01	Resistant	12.368359
TCGA-55-A48Z-01	Resistant	12.206624
TCGA-69-A59K-01	Resistant	11.674364
TCGA-71-6725-01	Resistant	12.955612
TCGA-86-8278-01	Resistant	12.898626
TCGA-86-8674-01	Resistant	12.269416
TCGA-95-A4VK-01	Resistant	12.126682
TCGA-NJ-A7XG-01	Resistant	12.084294
TCGA-05-4390-01	Sensitive	11.185142
TCGA-05-4427-01	Sensitive	12.808488
TCGA-05-4432-01	Sensitive	11.653808
TCGA-05-5428-01	Sensitive	11.353048
TCGA-44-8117-01	Sensitive	11.741016
TCGA-50-5941-01	Sensitive	12.535108
TCGA-50-8459-01	Sensitive	11.80382
TCGA-53-A4EZ-01	Sensitive	12.221865
TCGA-55-7576-01	Sensitive	12.758477
TCGA-55-7910-01	Sensitive	12.272837
TCGA-55-8505-01	Sensitive	12.474327
TCGA-55-8508-01	Sensitive	11.742415
TCGA-62-A46Y-01	Sensitive	12.092622
TCGA-62-A471-01	Sensitive	12.300653
TCGA-64-5779-01	Sensitive	11.720796
TCGA-64-5781-01	Sensitive	12.018607
TCGA-69-7973-01	Sensitive	12.036455
TCGA-73-4675-01	Sensitive	12.152646
TCGA-73-7498-01	Sensitive	12.34784
TCGA-86-7701-01	Sensitive	12.267404
TCGA-86-7711-01	Sensitive	12.443249
TCGA-86-7713-01	Sensitive	12.063998

TCGA-86-7955-01	Sensitive	12.298575
TCGA-86-8054-01	Sensitive	12.324566
TCGA-86-8075-01	Sensitive	12.142295
TCGA-86-8076-01	Sensitive	12.519486
TCGA-86-8279-01	Sensitive	11.62309
TCGA-86-8280-01	Sensitive	12.002579
TCGA-86-8669-01	Sensitive	12.718661
TCGA-86-8671-01	Sensitive	12.218158
TCGA-86-A4D0-01	Sensitive	12.286832
TCGA-86-A4P8-01	Sensitive	11.867893
TCGA-91-6848-01	Sensitive	11.98044
TCGA-91-7771-01	Sensitive	12.461088
TCGA-L9-A743-01	Sensitive	12.092156
TCGA-L9-A7SV-01	Sensitive	12.664432
TCGA-MP-A4T9-01	Sensitive	11.767531
TCGA-MP-A4TC-01	Sensitive	12.365385
TCGA-MP-A4TD-01	Sensitive	11.71146
TCGA-MP-A4TF-01	Sensitive	11.80014

* The gene expression values were quantified using RNA-Seq by Expectation-Maximization (RSEM) and batch-normalized to correct for technical variations. The data were generated from Illumina HiSeq RNA sequencing (RNASeqV2) and transformed using log2(TPM + 1) for normalization and comparability across samples.
